# Physical activity behaviours, knowledge, attitudes, and training needs among medical students in South Africa: a cross-sectional study

**DOI:** 10.1186/s12909-026-09933-x

**Published:** 2026-07-15

**Authors:** Mark Stoutenberg, Innocent Maposa, Georgia Torres

**Affiliations:** 1 Wolfson Research Institute for Health and Wellbeing, Durham University, Durham, UK; 2 Department of Sport and Exercise Sciences, Durham University, Durham, UK; 3 Department of Exercise Science and Sports Medicine, Faculty of Health Sciences, School of Therapeutic Sciences, University of the Witwatersrand, Johannesburg, South Africa; 4 Division of Epidemiology and Biostatistics, Department of Global Health, Faculty of Medicine and Health Sciences, Stellenbosch University, Stellenbosch, South Africa

**Keywords:** Africa, Curriculum, Exercise prescription, Medical education, Non-communicable diseases

## Abstract

**Background:**

While previous work has examined physical activity (PA) training in medical schools in high-income countries, little is known about PA training and related student attitudes and behaviours in low-and-middle income countries (LMICs). This study examined the PA knowledge, personal PA behaviours, attitudes toward PA promotion, and PA training needs of medical students at a major South African medical school across year of medical training.

**Methods:**

A paper-based questionnaire was administered in-person to medical students across all four years of the Graduate Entry Medical Programme (GEMP) between April and September 2021. The questionnaire assessed students’ personal PA behaviours, PA knowledge, attitudes toward PA promotion, and perceived PA training needs.

**Results:**

A total of 536 medical students participated with response rates highest among first-year students and lower in later-year cohorts following COVID-19 related disruptions. Medical student PA levels were low overall (34.8% met WHO PA recommendations) and generally declined with increasing year in medical school (GEMP 1 to 3). Nearly all students recognised the importance of PA in disease prevention (98.1%) and future patient care (95.0%); however, objective PA knowledge was low - only 9.1% and 5.2% of all medical students correctly reported WHO PA recommendations and demonstrated knowledge of core exercise prescription principles, respectively. Most students (82.3%) also desired greater PA training with the medical school curriculum.

**Conclusions:**

This study adds to the limited literature from LMIC settings demonstrating that medical students are interested in receiving PA training, recognise its importance in future clinical practice, but feel insufficiently prepared to counsel future patients.

**Supplementary Information:**

The online version contains supplementary material available at https://doi.org/10.1186/s12909-026-09933-x.

## Background

Non-communicable diseases (NCDs) are the leading cause of death globally, accounting for 41 million or 74% of deaths in 2020 [1]. The burden of NCDs is not shared equally across the world, as individuals in low- and middle-income countries (LMICs) have a 3.8 times higher rate of death due to cardiovascular disease than individuals in high-income countries [2]. In Africa, NCDs are responsible for 42% of all deaths with the rates ranging by country from 27% (Chad, Nigeria) to 88% (Mauritius) [3]. The increasing prevalence of NCDs in Africa is attributed to a range of interrelated factors, including lifestyle choices, genetic and epigenetic influences, and healthcare availability [4, 5]. NCDs cause an estimated 51% of all deaths in South Africa, most of which are due to cardiovascular disease and diabetes [6]. If not adequately addressed, the continued rise of NCDs will continue to cost millions of lives and trillions of dollars in the world economy [7].

Given this growing crisis, the World Health Organization (WHO) set a goal to reduce global mortality due to NCDs by 25% by the year 2025 [8]. To support this effort, the WHO identified a series of “best buys” for the prevention and management of NCDs [9], including strategies for reducing tobacco and alcohol use, unhealthy dietary practices, and physical inactivity. Globally, physical inactivity is estimated to be responsible for 7.2% and 7.6% of all-cause and cardiovascular deaths, respectively, with 28% of adults not achieving physical activity (PA) guidelines [10, 11]. Levels of physical inactivity in adults vary widely throughout Africa [12]. Data from a national South African survey conducted in 2012 estimated that 57.4% of individuals are physically inactive [13], although more recent nationally representative surveillance data remains limited. Barriers contributing to physical inactivity in South Africa have been attributed to limited access to recreational facilities, personal safety fears, and inadequate infrastructure for active living [14].

To overcome these barriers to PA, guidelines suggest engaging individuals through multi-sectoral approaches involving improvements to the built environment and transportation systems, changes in public policy, and community wide campaigns [15]. While a systems-based approach has the potential to influence change on a community level, complementary individualized strategies, such as PA counselling in health settings have also proven to be effective [16, 17]. Existing literature suggests that PA advice by health professionals has a small to moderate impact on increasing moderate-to-vigorous PA levels [17, 18], which, in turn, can have a significant impact on overall health outcomes. Despite the potential impact of providing PA counselling shown, physicians report feeling unprepared to counsel their patients due to inadequate knowledge and training [19, 20].

Effective PA counselling by future physicians likely depends on multiple interrelated factors, including medical students’ PA behaviours, their knowledge of PA guidelines and exercise prescription principles, their attitudes toward the importance of PA promotion in healthcare, and the broader educational and social environment in which medical training occurs. Understanding these domains collectively may help identify gaps in medical education and reveal opportunities for curriculum development. Previous studies have examined PA training and counselling in Canada [21], the United States [22, 23], Australia [24], and Europe [25, 26]; however, little is known about PA training in medical schools in Africa. Improving understanding of how PA can be integrated into medical school curricula in African settings may represent an important strategy for addressing the growing burden of NCDs. Thus, this study examined the PA personal behaviours, PA knowledge, attitudes towards PA promotion, and perceived PA training needs of South African medical students, as well as differences across year in medical school.

## Methods

### Overview

This study was conducted between April and September 2021 with medical students enrolled across all four years of the Graduate Entry Medical Programme (GEMP) in the Faculty of Health Sciences at the University of the Witwatersrand. A paper-based survey, which required less than ten minutes to complete, was administered in person to medical students during scheduled teaching activities across the 2021 academic year.

### Participants

The study population included all full-time medical students enrolled in GEMP years 1–4 at the University of the Witwatersrand during the 2021 academic year. At the University of the Witwatersrand, the GEMP represents the final four years of the six-year Bachelor of Medicine and Bachelor of Surgery (MBBCh) programme. Students may enter this training through either a traditional six-year entry pathway or a graduate-entry pathway following completion of a prior degree. For consistency with institutional terminology, students were grouped according to their GEMP year of study. The total number of enrolled students and response rates for each GEMP year are presented in Supplementary Table 1.

### Questionnaire development

A paper-based questionnaire was developed from previous studies examining PA knowledge, attitudes, and counselling preparedness among medical students in the United States [22] and the United Kingdom [25]. The questionnaire (Supplemental Table 2) consisted of five sections: demographic information, personal PA behaviours, PA knowledge, attitudes toward PA promotion, and PA education in medical school, including perceptions of institutional and peer support for PA participation. Self-reported PA levels were collected using the PA Vital Sign [27], a clinical tool that quickly and accurately determines whether an individual is meeting global PA guidelines [28], as well as single items about their weekly strength training practices and sedentary behaviour. The PA Vital Sign was administered using wording consistent with previously published applications of the tool, which demonstrated acceptable validity and reliability as a brief clinical PA screening instrument [29–31]. PA knowledge was evaluated using short answer questions that asked about the aerobic and strength training components of the global PA guidelines [28], as well as the core components of an exercise prescription (FITT: frequency, intensity, time, type). Medical students’ attitudes towards PA were assessed using 5-point Likert scales that assessed their perceived importance of PA in future clinical work, perceived PA knowledge, and ability to assist patients become more physically active. Lastly, 5-point Likert scales were used to query students about PA promotion on campus, the PA attitudes of their peers, and their general attitudes towards being active and fit.

The questionnaire was piloted by ten medical school faculty members to assess clarity, contextual appropriateness, and comprehensibility prior to final administration. Major modifications to the questionnaire were intentionally avoided to preserve comparability with prior work across different settings, and pilot testing did not identify the need for substantive changes to the questionnaire items.

### Procedures for survey tool completion

GEMP I students completed the paper-based questionnaire prior to a series of COVID-19-related campus closures. Students were provided with the questionnaire and asked to complete it prior to a practical session at the Department of Exercise Science and Sports Medicine. These sessions included training on the measurement of cardiovascular disease risk factors as part of health assessments, such as blood pressure, resting heart rate, waist circumference, PA levels, cardio-respiratory fitness, and muscle strength. Students in GEMP years 2–4 completed the questionnaire during small group (8–10 students), in-person tutorial sessions in the early phases of campus re-opening in late 2021. Although data collection occurred between April and September 2021, intermittent COVID-19-related restrictions and phased return-to-campus policies remained in place within South African universities during this period, resulting in differing opportunities for in-person survey administration across GEMP cohorts.

The research team was provided with time at the beginning of tutorials during which students could choose to complete the questionnaire. Tutorial attendance was not compulsory in order to accommodate students who preferred to continue studying online during the uncertainty surrounding campus re-opening and COVID-19 health concerns; thus, not all medical students in GEMP years 2–4 were present to participate. As part of the study design, the questionnaire was developed with the intention of supporting potential follow-up phases of data collection, including repeated survey administration and longitudinal tracking of students across GEMP years. Therefore, student names and identification numbers were initially collected to prevent duplicate responses and facilitate future linkage of responses over time. Following data collection, all participant responses were coded and de-identified prior to analysis, and password-protected linking document was stored separately and securely.

### Data analysis

Data were entered into an Excel spreadsheet and exported to STATA version 17 (StataCorp. 2021. Stata Statistical Software: Release 17. College Station, TX: StataCorp LLC.) for preprocessing and analysis. Categorical variables were summarized using frequencies and percentages, while continuous variables were described using either medians and interquartile range or means and standard deviations, as appropriate. Students were classified as meeting WHO PA recommendations if their self-reported moderate-to-vigorous PA equalled or exceeded 150 min per week, based on responses to the PA Vital Sign items. Likert-scale items were scored on a 5-point scale, with higher scores indicating greater levels of agreement.

For logistic regression analyses, all Likert-scale variables were dichotomised into non-agreement (strongly disagree, disagree, and neutral = 0) and agreement (agree and strongly agree = 1). Odds ratios therefore represent the odds of agreement with the outcome statement. Fisher’s Exact tests were used to assess bivariate associations between categorical variables. Independent Student’s t-tests were used to compare normally distributed continuous variables between two groups, while Mann-Whitney U tests were used for non-normal continuous variables. Normality was assessed only for continuous variables using graphical inspection and distributional characteristics. Individual Likert-scale items were treated as ordinal variables and were therefore not assessed for normality. Parametric or non-parametric statistic tests were selected accordingly. Univariate and multivariable logistic regression models were used to assess factors associated with: (1) confidence in PA counselling, and (2) the desire for additional PA training. Backward stepwise variable selection was then performed using an elimination criterion of *p* > 0.20, with variables sequentially removed from the multivariable model if they exceeded this threshold. Year in medical school was retained in the final model as an a priori variable of interest based on the study objectives, while gender was retained to account for potential confounding, regardless of statistical significance. Univariate regression analyses were performed for descriptive purposes and were not used to determine inclusion of variables in the multivariable models.

### Ethics

Ethical approval was provided by the Human Research Ethics Committee (Medical) at the University of the Witwatersrand [Ref. M191043] for all study materials and procedures. A written informed consent form was attached to the beginning of the questionnaire and was obtained from all participants prior to beginning the questionnaire. Participation in the study was voluntary; if a student did not consent to participate in the study, they were advised not to continue to the questionnaire. Paper-based questionnaires containing identifiable information were stored in locked filing cabinets in the primary investigator’s office. Following data collection, participant responses were coded and entered in an electronic database for analysis. A separate electronic linking document containing identifiable information was stored securely in a password-protected folder accessible only to the primary investigator.

## Results

A total of 412 medical students completed the questionnaire, representing an overall response rate of 38.5%. Response rates were highest among GEMP 1 students (68.1%) and lower among students in GEMP years 2–4 following COVID-19-related disruptions to in-person teaching activities and tutorial attendance. Participant demographic characteristics are presented in Table 1, while total cohort enrolment numbers and response rates by GEMP year are provided in Supplementary Table 1.

**Table 1:** Demographic characteristics of participating medical students

	Overall (*n* = 536)	GEMP Year 1 (*n* = 274)	GEMP Year 2 (*n* = 80)	GEMP Year 3 (*n* = 91)	GEMP Year 4 (*n* = 91)	*p*-value
Age, Median (IQR)	22 (21–24)	21 (20–22)	22 (21–23)	23 (22–24)	25 (24–26)	**< **0.001^**+**^
Gender						0.95^£^
Male	175 (32.8%)	92 (33.7%)	24 (30.0%)	30 (33.3%)	29 (32.2%)	
Female	358 (67.2%)	181 (66.3%)	56 (70.0%)	60 (66.7%)	61 (67.8%)	
Country of Origin						0.039^¥^
Non-South African	32 (6.1%)	17 (6.3%)	9 (11.5%)	5 (5.7%)	1 (1.1%)	
South African	493 (93.9%)	254 (93.7%)	69 (88.5%)	82 (94.3%)	88 (98.9%)	
Ethnicity						0.626^¥^
Caucasian	179 (33.5%)	100 (36.6%)	24 (30.0%)	27 (29.7%)	28 (30.8%)	
African	219 (40.9%)	109 (39.9%)	32 (40.0%)	36 (39.6%)	42 (46.2%)	
Indian	107 (20.0%)	47 (17.2%)	21 (26.3%)	21 (23.1%)	18 (19.8%)	
Coloured	22 (4.1%)	11 (4.0%)	2 (2.5%)	6 (6.6%)	3 (3.3%)	
Other	8 (1.5%)	6 (2.2%)	1 (1.2%)	1 (1.1%)	0 (0.0%)	
Adherence to WHO PA recommendations						0.011^£^
No	349 (65.2%)	168 (61.5%)	58 (72.5%)	70 (76.9%)	53 (58.2%)	
Yes	186 (34.8%)	105 (38.5%)	22 (27.5%)	21 (23.1%)	38 (41.8%)	
Time spent seated per day						< 0.001^¥^
0–5 h	53 (10.1%)	15 (5.5%)	1 (1.3%)	16 (17.8%)	21 (23.1%)	
6–10 h	241 (45.7%)	135 (49.8%)	37 (49.3%)	36 (40.0%)	33 (36.3%)	
11–15 h	123 (23.3%)	80 (29.5%)	16 (21.3%)	11 (12.2%)	16 (17.6%)	
16–20 h	37 (7.0%)	11 (4.1%)	9 (12.0%)	5 (5.6%)	12 (13.2%)	
Unsure	73 (13.9%)	30 (11.1%)	12 (16.0%)	22 (24.4%)	9 (9.9%)	

Data are presented as median (IQR) for continuous measures and n (%) for categorical measures

*PA* physical activity, *WHO* World Health Organization, *GEMP* Graduate Entry Medical Programme

^+^ *p*-value computed using Kruskal Wallis test to assess differences in age distribution across year of medical school

^£^ *p*-value computed using Pearson’s χ^2^ test to assess association between year of medical school and the characteristic

^¥^ *p*-value computed using Fishers’ Exact test to assess association between year of medical school and the characteristic

### Medical student PA behaviours

Slightly more than one third of all medical students reported PA levels meeting WHO PA recommendations; however, students in GEMP 2 and 3 were significantly less likely than their classmates to meet these guidelines (27.5% and 23.1%, respectively). Reported adherence to WHO PA recommendations declined from GEMP 1 to 3 (38.5% to 23.1%) before increasing again among students in GEMP 4 (41.8%). Nearly half (45.7%) of the medical students reported sitting an average of 6–10 h per day. Time spent seated increased significantly across year in medical school, with students in GEMP year 3 reporting the greatest sitting time (*p* < 0.001).

### Medical student PA knowledge and preparedness for counselling

Medical students reported moderate levels of confidence in their knowledge to provide PA counselling, with 38.1% strongly agreeing or agreeing that they possessed sufficient knowledge to properly advise patients on PA (Supplementary Table 3). However, objective knowledge of PA recommendations and exercise prescription principles were low across all cohorts (Table 2). Students in GEMP years 3 and 4 were significantly more likely to report sufficient knowledge to provide PA counselling compared with students in earlier cohorts (*p* = 0.003; Table 3). Overall, only 9.1% of students correctly identified WHO PA recommendations, while 5.2% correctly identified the core FITT principles required for exercise prescription.

**Table 2:** Medical student physical activity knowledge

	Overall (*n* = 536)	Year 1 (*n* = 274)	Year 2 (*n* = 80)	Year 3 (*n* = 91)	Year 4 (*n* = 91)	*p*-value*
Desire to change own exercise habits						0.92
Yes	468 (87.5%)	238 (86.9%)	71 (89.9%)	79 (86.8%)	80 (87.9%)	
No	67 (12.5%)	36 (13.1%)	8 (10.1%)	12 (13.2%)	11 (12.1%)	
Knowledge of WHO PA requirements						< 0.001
Yes	49 (9.1%)	43 (15.7%)	0 (0.0%)	6 (6.6%)	0 (0.0%)	
No	487 (90.9%)	231 (84.3%)	80 (100.0%)	85 (93.4%)	91 (100.0%)	
Correct identification of all principles of an exercise Rx**						< 0.001
Yes	28 (5.2%)	4 (1.5%)	1 (1.2%)	21 (23.1%)	2 (2.2%)	
No	508 (94.8%)	270 (98.5%)	79 (98.8%)	70 (76.9%)	89 (97.8%)	

*PA* physical activity, *Rx* prescription, *WHO* World Health Organization

* All *p*-values represent Fishers’ Exact test of association

** Correctly identifying the core concepts of an exercise prescription as frequency, intensity, time, and type (FITT)

**Table 3:** Medical student attitudes towards physical activity training and promotion with patients by year in medical school

	Overall (*n* = 536)	Year 1 (*n* = 274)	Year 2 (*n* = 80)	Year 3 (*n* = 91)	Year 4 (*n* = 91)	*p*-value
Confidence in changing own personal exercise habits in next 6 months*	6.65 (2.21)	6.80 (2.10)	6.73 (2.04)	5.90 (2.47)	6.90 (2.28)	0.010
Prevention is less interesting than treatment	2.01 (1.17)	2.01 (1.20)	2.13 (1.17)	2.02 (1.13)	1.87 (1.14)	0.54
Importance of PA as a treatment and prevention tool for disease	4.68 (0.57)	4.65 (0.52)	4.81 (0.39)	4.69 (0.69)	4.67 (0.70)	0.17
Promoting PA is an important part of their future patients consults	4.49 (0.64)	4.45 (0.61)	4.63 (0.54)	4.56 (0.76)	4.48 (0.69)	0.12
Students have sufficient knowledge to properly advise PA	3.18 (0.96)	3.14 (0.98)	2.91 (0.93)	3.37 (0.91)	3.36 (0.86)	0.003
Belief they can effectively help patients increase PA levels	3.60 (0.79)	3.54 (0.79)	3.51 (0.75)	3.82 (0.75)	3.64 (0.84)	0.019
Would like more training in PA	4.08 (0.85)	4.06 (0.88)	4.18 (0.81)	4.07 (0.90)	4.07 (0.74)	0.75
Medical school encourages us to exercise	2.69 (1.04)	2.68 (1.07)	2.53 (1.06)	2.81 (1.03)	2.70 (0.89)	0.38
Classmates encourage each other to exercise	2.92 (0.94)	2.85 (0.93)	2.62 (0.90)	3.00 (0.95)	3.30 (0.92)	< 0.001
Provide more effective counselling if students exercise and stay fit	4.03 (0.75)	3.99 (0.78)	4.10 (0.71)	4.06 (0.80)	4.08 (0.66)	0.58

*PA* physical activity

* Confidence in changing own personal exercise habits in the next 6 months was assessed using a 1–10 scale, with 1 indicating “very confident” and 10 indicating “not confident at all.” All remaining items were assessed using 5-point Likert scales, with 1 indicating “strongly disagree” and 5 indicating “strongly agree.” Values are presented as mean (standard deviation). *p-value* denotes significant differences between medical students across year of medical school

### Medical students’ attitudes toward PA promotion

Nearly all medical students strongly agreed or agreed on the importance of promoting PA for disease prevention and treatment (98.1%), promoting it with their future patients (95.0%), and desired additional PA training in the medical education curriculum (82.3%) (Supplementary Table 3). However, fewer medical students strongly agreed or agreed that their classmates (26.3%) and medical school (21.9%) encouraged them to be physically active. Table 3 presents medical student attitudes towards PA and promoting it with their future patients by year in medical school. Medical students in their final years of medical school reported being significantly more confident in being able to effectively counsel patients to increase their PA levels (*p* = 0.019). There were significant increases in medical student confidence in changing their personal exercise habits in the future (*p* = 0.010), having sufficient knowledge to properly advise future patients on PA (*p* = 0.003), believing that they can effectively help patients increase their PA levels (*p* = 0.019), and that their classmates encouraged each other to exercise (*p* < 0.001).

### Predictors of PA counselling preparedness and training needs

Univariate logistic regression analyses identified several factors associated with medical students’ confidence in their ability to effectively help patients increase PA levels. In the multivariate logistic regression model (Table 4), significant factors associated with greater confidence in PA counselling included being in GEMP year 3 (*p* = 0.010), adherence to WHO PA recommendations (*p* = 0.017), believing in the importance of PA as treatment (*p* = 0.017), possessing sufficient PA knowledge (*p* < 0.001), and believing that exercising and staying fit would improve credibility and effective counselling (*p* = 0.033).

**Table 4:** Factors associated with student belief that they can effectively help patients increase their physical activity

Characteristics	Univariate logistic regression		Multivariable logistic regression*	
	OR [95% CI]	*p*-value	aOR [95% CI]	*p*-value
Gender				
Male	1			
Female	0.62 [0.43–0.91]	0.014	0.70 [0.44–1.13]	0.144
Year in Medical School				
1	1			
2	0.87 [0.53–1.43]	0.581	0.84 [0.46–1.54]	0.570
3	2.18 [1.30–3.66]	0.003	2.31 [1.22–4.37]	0.010
4	1.21 [0.75–1.95]	0.445	0.94 [0.52–1.69]	0.835
Adherence to WHO PA recommendations				
No	1			
Yes	2.60 [1.77–3.82]	< 0.001	1.84 [1.11–3.05]	0.017
Desire to change PA habits				
No	1			
Yes	0.47 [0.26–0.83]	0.009	†	†
Prevention less interesting	0.99 [0.86–1.15]	0.920	-	-
Importance of PA as treatment	2.22 [1.57–3.14]	< 0.001	1.64 [1.03–2.60]	0.017
Students have sufficient PA knowledge	3.09 [2.44–3.92]	< 0.001	2.65 [2.03–3.48]	< 0.001
Medical School encourages PA	1.26 [1.06–1.49]	0.008	-	-
Classmates encourage each other to exercise	1.23 [1.02–1.48]	0.028	-	-
Provide more credible counselling if fit	1.77 [1.38–2.26]	< 0.001	1.40 [1.03–1.90]	0.033
Confident can change exercise habit in 6 months	1.16 [1.06–1.26]	0.001	1.06 [0.95–1.18]	0.332

*aOR* adjusted odds ratios, *OR* odds ratios, *PA* physical activity, *WHO* World Health Organization

* Backward stepwise variable selection was performed from an initial multivariable model containing all candidate covariates. Variables with *p* > 0.20 were sequentially removed during model fitting, except for year in medical school and gender, which were retained in the final model a priori in the final model. Univariate analyses were not used for variable selection. Variables without adjusted odds ratios were removed during backward elimination

† Parameter not estimable in the multivariable model. Odds ratios (ORs) > 1 indicate greater likelihood of reporting confidence in helping patients increase their physical activity levels

In the second multivariable logistic regression model, believing in the importance of PA as treatment (*p* = 0.025) and reporting sufficient PA knowledge (*p* = 0.029) were the only two variables that remained significantly associated with medical students’ desire for additional PA training (Table 5).

**Table 5:** Factors associated with medical students desiring more training in physical activity

Characteristics	Univariate logistic regression		Multivariable logistic regression*	
	OR [95% CI]	*p*-value	aOR [95% CI]	*p*-value
Gender				
Male	1			
Female	1.07 [0.67–1.71]	0.783	1.24 [0.70–2.21]	0.456
Year in Medical School				
1	1			
2	0.52 [0.25–1.07]	0.077	1.53 [0.66–3.52]	0.318
3	0.61 [0.32–1.17]	0.135	2.08 [0.90–4.79]	0.087
4	0.61 [0.32–1.17]	0.135	1.52 [0.71–3.28]	0.282
Adherence to WHO PA recommendations				
No	1			
Yes	1.06 [0.66–1.69]	0.807	-	-
Desire to change PA habits				
No	1			
Yes	3.17 [1.81–5.56]	< 0.001	†	†
Prevention less interesting	0.80 [0.67–0.96]	0.015	0.83 [0.67–1.02]	0.080
Importance of PA as treatment	1.69 [1.20–2.37]	0.003	1.70 [1.07–2.71]	0.025
Students have sufficient PA knowledge	0.86 [0.68–1.09]	0.208	0.74 [0.51–0.97]	0.029
Medical school encourages PA	1.10 [0.89–1.37]	0.372	-	-
Classmates encourage each other to exercise	1.10 [0.87–1.39]	0.445	-	-
Provide more credible counselling if fit	1.60 [1.21–2.13]	0.001	1.43 [0.99–2.05]	0.056
Confident can change exercise habit in 6 months	1.12 [1.00-1.25]	0.042	1.11 [0.99–1.26]	0.085

*aOR *adjusted odds ratio, *OR* odds ratio, *PA* physical activity, *WHO* World Health Organization

* Backward stepwise variable selection was performed from an initial multivariable model containing all candidate covariates. Variables with *p* > 0.20 were sequentially removed during model fitting, except for year in medical school and gender, which were retained in the final model a priori in the final model. Univariate analyses were not used for variable selection. Variables without adjusted odds ratios were removed during backward elimination

† Parameter not estimable in the multivariable model. Odds ratios (ORs) > 1 indicate greater odds of reporting a desire for additional PA training

## Discussion

This study examined the PA knowledge, personal PA behaviours, attitudes toward PA promotion, and PA training needs of South African medical students across the four years of medical school. Given the growing burden of NCDs in Africa and the low-cost preventive potential of PA promotion, understanding how medical students perceive and engage with PA training is particularly important. To our knowledge, the inclusion of PA in medical schools within Africa and other LMIC setting has not been rigorously examined. Overall, medical students demonstrated strong recognition of the importance of PA and a clear desire for greater PA training within the curriculum; however, substantial gaps remained in knowledge of PA guidelines and exercise prescription principles. Students also perceived limited institutional and peer support for engaging in PA.

### Medical student physical activity knowledge and training needs

Similar to previous studies in high income countries, such as the United States [23, 32], Australia [24], and the United Kingdom [33], there continues to be a persistent lack of PA training in medical schools, despite medical student awareness of the importance of PA in the overall health of their patients and in non-communicable disease prevention [34, 35]. This lack of PA training is often accompanied by an overall limited PA knowledge. Only 22% of students in a Brazilian medical school knew the WHO recommendation for weekly aerobic activity [36], and less than 1% of students at a U.S. osteopathic medical school correctly identified national PA recommendations [37]. In the current study, just over a third of medical students felt they had a sufficient level of PA knowledge, while only 9.1% and 5.2% correctly identified WHO PA guidelines and the core principles of developing an exercise prescription.

Despite these gaps, medical students strongly valued PA and desired greater training. In the current study, 82.3% of students agreed or strongly agreed that they desired more PA training as part of their program. This aligns with results from studies in Brazil [36], Scotland [38], and the United States [32], in which 74.5%, > 80%, and 84.7% of students desired greater PA training in their medical school curriculum, respectively. These findings are not unique to medical education; a greater desire for more PA training has also been identified among future nurse practitioners [39], physiotherapists [40], and pharmacists [41]. Moreover, medical student PA knowledge and attitudes (e.g., desire for greater training) are inextricably linked; in our study, valuing the importance of PA as important in the treatment of disease was a significant predictor of students desiring more PA training in their curriculum.

### Individual PA behaviours and PA promotion attitudes

Beyond formal curriculum exposure, students’ personal PA behaviours may also influence their future counselling practices. Maintaining an active lifestyle during medical training is associated with academic success and achievement [42, 43], as well as improved levels of physical and mental health [22, 44, 45]. Despite the heavy demands of medical training, several studies report that medical students are more likely to meet PA recommendations than the general population [21, 22, 37, 46]. Medical students in the U.S. who met PA recommendations were more likely to recognise that physician PA behaviours influence patient perceptions and improve their credibility in providing effective PA counselling [22]. Similarly, active students demonstrated positive attitudes and were more likely to counsel patients on PA [21, 22]. In the current study, however, medical student self-reported PA levels were lower than population averages, with only 34.8% meeting PA guidelines for aerobic activity. These findings are consistent with a previous study at the same medical school in which only 30% of students met WHO PA requirements and were below population cardiorespiratory fitness norms [47].

The disconnect between positive attitudes towards PA promotion and student personal behaviours has been observed in several previous studies and may reflect the competing academic demands, sedentary study patterns, and time constraints associated with medical training. Across the four years of medical school, PA levels generally declined between the earlier and later years of training. Given the cross-sectional design and lower response rates observed in later GEMP cohorts, these findings should be interpreted cautiously and may partially reflect sampling variability or nonresponse bias. Nevertheless, the decline in PA levels observed from the first to the third year is similar to Frank et al. [22] who reported that medical students perceived PA counselling to be more relevant to their intended practices upon entry to medical school compared to their final year. One possible explanation is that the latter years of medical school involve greater time spent in clinical rotations, often with supervisors who are not trained nor feel competent themselves to provide PA counselling with patients [48].

### The medical school environment and PA promotion

One explanation for the low levels of self-reported PA observed in the current study may be the medical school environment. The medical school environment influences not only the delivery of PA-related content, but also the formation of positive lifestyle habits. Previous research suggests that medical students value faculty role modelling of healthy lifestyles and that encouragement from classmates and medical school staff faculty increase the relevance of PA counselling [22]. Yet, in the current study, only 21.9% and 26.3% of the medical students felt that their medical school and classmates, respectively, encouraged them to be active. Although perceptions of support for PA improved across the medical school years, these findings suggest that further improvements could be made in how the medical school environment values and supports student PA. Beyond curricular changes, ensuring sufficient extracurricular opportunities, appropriate facilities, and a supportive institutional culture all appear to be important factors in the development of positive attitudes (e.g., self-efficacy) and healthy lifestyle habits among medical school students [32, 48].

### Strengths and limitations

This study has several limitations. First, there was a significant decline in the medical student response rate between the GEMP 1 and GEMP 2–4 cohorts. This difference largely reflects the timing of data collection, as GEMP 1 students completed the survey prior to COVID-19-related campus restrictions and reductions in in-person educational activities, whereas surveys were collected with other cohorts after resuming classes. Because demographic information was not available for students who did not participate, it was not possible to assess the representativeness of the sample or the potential impact of nonresponse bias. The lower response rates observed in the later GEMP cohorts may also have introduced nonresponse bias and should be considered when interpreting differences observed across years of medical school. Future studies conducted under more typical educational conditions and using broader recruitment approaches may help improve participation across all year groups. Second, this study presents data from a cross-sectional sample of students across four years of medical school. While initial conclusions may be drawn regarding differences between cohorts of students as they progress through medical school, these findings would be strengthened by following cohorts of students longitudinally throughout their medical training. Lastly, this study was conducted at a single medical school, limiting its generalizability to medical schools in South Africa.

At the same time, these limitations are offset by several strengths. To our knowledge, this is the first study to comprehensively examine medical students’ PA knowledge, personal PA behaviours, attitudes towards PA promotion, and perceived PA training needs within a medical school in South Africa. Additionally, this study included students across all four years of the GEMP, allowing comparison of these domains across different stages of medical training. Additionally, the questionnaire was adapted from instruments previously used internationally, supporting comparability with prior literature while remaining contextually appropriate for the South African medical education setting. Finally, this work contributes important data from an LMIC context, where evidence regarding PA training in undergraduate medical education remains limited.

### Future recommendations

Several studies have provided guidance for the training of medical students and health professionals that may serve as models for increasing PA content in medical school curricula [49–52]. Internationally, PA has been integrated into medical education through a range of approaches, including broader health promotion or lifestyle medicine interventions [53], PA-specific curricular interventions [54], and self-directed educational tools [55]. Beyond curricular reform, additional strategies may include developing cross-disciplinary partnerships with other health disciplines (e.g., exercise science/kinesiology programs) to provide expertise on PA and exercise topics [48], incorporating rotations in PA laboratories or fitness centres [56], and involving medical students in teaching PA to the local community as part of a service-learning experiences [57]. Ultimately though, for PA to achieve sustained integration within medical education, progress toward its inclusion in national licensing examinations will likely be necessary [48, 58].

## Conclusions

This study, conducted at a large urban medical training institution in South Africa, provides insight into medical student PA personal behaviours, attitudes towards PA promotion, and PA training needs, adding to the growing body of literature supporting the inclusion of PA training in the medical school curricula. Similar to studies conducted in higher income countries, our findings demonstrate that medical students recognise the importance of PA in future clinical practice and desire greater PA training, yet feel unprepared to counsel future patients. Although differences were observed across the four years of medical training, objective knowledge of PA guidelines and exercise prescription principles remained low across all cohorts. Our findings also highlight the potential importance of the medical school environment, beyond curricular changes, in promoting healthy lifestyle behaviours and supporting medical students to engage in PA during their training. We hope this work contributes to ongoing efforts to strengthen the inclusion of PA training within medical school curricula and national licensing examinations around the world.

## Supplementary Information

Supplementary Material 1.

Supplementary Material 2.

Supplementary Material 3.

## Data Availability

The dataset(s) supporting the conclusions of this article is(are) available upon request from the corresponding author.

## Abbreviations

GEMP: Graduate Entry Medical Programme
LMICs: Low- and middle-income countries
NCDs: Non-communicable diseases
PA: Physical activity
WHO: World Health Organization
