# Supplementary material for: Physical activity behaviours, knowledge, attitudes, and training needs among medical students in South Africa: a cross-sectional study
Source: BMC Med Educ. 2026 Jul 15;26:1523. doi: 10.1186/s12909-026-09933-x (PMC13602619; doi:10.1186/s12909-026-09933-x)
Supplement: Supplementary file 1 [file 12909_2026_9933_MOESM2_ESM.pdf]

CONSENT:
I confirm that the procedures of this study have been thoroughly explained to me. I acknowledge that the information collected will remain strictly confidential and may be used for research purposes, but no reference to my name will be revealed in any publication or statistical analysis.

DATE: ☐ **NAME:** ☐

SIGNATURE : ☐

Please read the attached information sheet and sign the consent form before you fill out the questionnaire. By answering the questionnaire, you consent to participate in the study. Please answer all of the following questions. You may choose more than one response where applicable. Mark your response with a X in the appropriate box(es).

Date:

Demographic information:

1. **Study number:** ____________ **Student number:** ____________
2. **Gender:** ☐ Male ☐ Female
3. **Age:** ___________
4. **GEMP year of study:**

Year 1 ☐ Year 2 ☐ Year 3 ☐ Year 4 ☐

5. **Ethnicity:**

Caucasian ☐ African ☐ Indian ☐ Coloured ☐ Other: _____________

6. **Country of origin:**

South Africa: _______________________

Other:……………………………… (state)

PHYSICAL ACTIVITY

Physical Activity Knowledge

For the following questions, physical activity refers to any activity that one does from low intensity (walking) to vigorous intensity (playing competitive sports).

7. How many minutes of moderate intensity aerobic activity should an adult do per week to gain health benefits?

   ______ minutes/week

8. How many minutes of vigorous intensity aerobic activity should an adult do per week to gain health benefits?

   ______ minutes/week

9. How many days per week should strength/resistance exercise be performed? (e.g., using weights or doing squats).

   ______ days/week

10. What would you include in the prescription of physical activity for a patient?

    ____

    ____

    ____

Attitudes Towards Physical Activity

Please mark with an "X" the response that most closely fits your feelings towards the statement given. Responses are on a scale from Strongly agree to Strongly disagree.

11. Physical activity is an important treatment and prevention tool for disease.

☐	☐	☐	☐	☐
Strongly agree	Agree	Neutral	Disagree	Strongly disagree

12. Promoting physical activity is an important part of my current/future patient consults.

☐	☐	☐	☐	☐
Strongly agree	Agree	Neutral	Disagree	Strongly disagree

13. I have sufficient knowledge to properly advise people about physical activity.

☐	☐	☐	☐	☐
Strongly agree	Agree	Neutral	Disagree	Strongly disagree

14. I believe that I can effectively help my patients increase their physical activity levels.

☐	☐	☐	☐	☐
Strongly agree	Agree	Neutral	Disagree	Strongly disagree

15. I would like more training in physical activity

☐	☐	☐	☐	☐
Strongly agree	Agree	Neutral	Disagree	Strongly disagree

Your Personal Physical Activity Practices

16. How many minutes of moderate intensity aerobic activity do you do per week?

_______ minutes/week

17. How many minutes of vigorous intensity aerobic activity do you do per week?

_______ minutes/week

18. How many days per week do you perform strength/resistance exercise? (e.g., using weights or doing squats).

_______ days/week

19. This question is in regards to the time you spend sitting during the last 7 weekdays. Include time spent at work, at home, while doing course work and during leisure time. This may include time spent sitting at a desk, visiting friends, reading, or sitting or lying down to watch television.

20. During the last 7 days, how much time did you spend sitting on a weekday?

______ hours per day

______ minutes per day

______ Not sure

21. On a scale of 1 -10, during the next 6 months, I'm extremely confident I can change my exercise habits.

Not at all Confident					Neutral					Very Confident
0	1	2	3	4	5	6	7	8	9	10

☐ I do not wish to change my exercise habits

Physical Activity Education in Medical School

22. My medical school encourages students to exercise.

☐	☐	☐	☐	☐
Strongly agree	Agree	Neutral	Disagree	Strongly disagree

23. My classmates encourage each other to exercise.

☐	☐	☐	☐	☐
Strongly agree	Agree	Neutral	Disagree	Strongly disagree

24. I will be able to provide more credible and effective counselling, if I exercise and stay fit.

☐	☐	☐	☐	☐
Strongly agree	Agree	Neutral	Disagree	Strongly disagree

25. Prevention is less interesting to me than treatment.

☐	☐	☐	☐	☐
Strongly agree	Agree	Neutral	Disagree	Strongly disagree
