# Supplementary material for: Physical activity behaviours, knowledge, attitudes, and training needs among medical students in South Africa: a cross-sectional study
Source: BMC Med Educ. 2026 Jul 15;26:1523. doi: 10.1186/s12909-026-09933-x (PMC13602619; doi:10.1186/s12909-026-09933-x)
Supplement: Supplementary file 2 [file 12909_2026_9933_MOESM1_ESM.docx]

**Supplemental Table 2. Medical student response rate across year in medical school.**

| **Year of Programme** | **# of Enrolled Students** | **# of Respondents** | **Response Rate (%)** |
| --- | --- | --- | --- |
| GEMP 1 | 450 | 274 | 60.9% |
| GEMP 2 | 420 | 80 | 19.0% |
| GEMP 3 | 372 | 91 | 24.5% |
| GEMP 4 | 320 | 91 | 28.4% |

GEMP: Graduate Entry Medical Programme
