# Supplementary material for: Physical activity behaviours, knowledge, attitudes, and training needs among medical students in South Africa: a cross-sectional study
Source: BMC Med Educ. 2026 Jul 15;26:1523. doi: 10.1186/s12909-026-09933-x (PMC13602619; doi:10.1186/s12909-026-09933-x)
Supplement: Supplementary file 3 [file 12909_2026_9933_MOESM3_ESM.docx]

**Supplemental Table 3. Overall level of medical student agreement towards physical activity training and promotion with patients.**

| **Item** | **Strongly Agree** | **Agree** | **Neutral** | **Disagree** | **Strongly Disagree** |
| --- | --- | --- | --- | --- | --- |
| Importance of PA as a treatment and prevention tool for disease | 385 (71.8%) | 141 (26.3%) | 6 (1.1%) | 0 (0%) | 4 (0.7%) |
| Promoting PA is an important part of their future patients consults | 301 (56.2%) | 208 (38.8%) | 23 (4.3%) | 1 (0.2%) | 3 (0.6%) |
| Students have sufficient knowledge to properly advise PA | 40 (7.5%) | 164 (30.6%) | 204 (38.1%) | 110 (20.5%) | 18 (3.4%) |
| Provide more effective counselling if I exercise and stay fit | 132 (24.8%) | 308 (57.9%) | 73 (13.7%) | 15 (2.8%) | 4 (0.8%) |
| Prevention is less interesting than treatment | 32 (6.0%) | 39 (7.4%) | 58 (10.9%) | 173 (32.6%) | 228 (43.0%) |
| Belief they can effectively help patients increase PA levels | 57 (10.6%) | 254 (47.4%) | 181 (33.8%) | 43 (8.0%) | 1 (0.2%) |
| Would like more training in PA | 172 (32.1%) | 269 (50.2%) | 70 (13.1%) | 15 (2.8%) | 10 (1.9%) |
| Medical school encourages us to exercise | 18 (3.4%) | 98 (18.5%) | 189 (35.6%) | 151 (28.4%) | 75 (14.1%) |
| Classmates encourage each other to exercise | 19 (3.6%) | 121 (22.7%) | 227 (42.7%) | 127 (23.9%) | 38 (7.1%) |
